# Physical activity initiated by employer and its health effects; an eight week follow-up study

**DOI:** 10.1186/s12889-016-3035-8

**Published:** 2016-05-04

**Authors:** Marit Skogstad, Lars-Kristian Lunde, Øivind Skare, Asgeir Mamen, Jose Hernán Alfonso, Reidun Øvstebø, Bente Ulvestad

**Affiliations:** Department of Occupational Medicine and Epidemiology, National Institute of Occupational Health, Box 8149 Dep, 0033 Oslo, Norway; Department for Work Psychology and Physiology, National Institute of Occupational Health, Box 8149 Dep, 0033 Oslo, Norway; Kristiania University College, Norwegian School of Health Sciences, Box 1190 Sentrum, 0107 Oslo, Norway; The R&D Group, Department of Medical Biochemistry and Clinical Pharmacology, Oslo University Hospital, Ullevaal, Norway

**Keywords:** Employer initiated physical activity, Maximal oxygen uptake, Blood pressure, Resting heart rate, Blood lipids

## Abstract

**Background:**

While the health benefits of physical activity are well established, little is known about health effects of physical activity programs initiated by employer.

**Methods:**

Background data and level of physical activity were collected by questionnaire among 78 men and 43 women working in road maintenance pre and post an 8-week physical activity motivational program. As a part of the program steps measured by accelerometer were registered online where team and individual performances could be continuously monitored. The physical activity levels were registered as 1) those physical active ≤1 time per week, 2) 2–3 times per week and 3) ≥4 times a week. Maximal oxygen uptake (VO_2max_), blood pressure, resting heart rate (RHR) and blood samples (glycosylated hemoglobin, lipids and C-reactive protein) were obtained at baseline and after eight weeks. Mixed models were applied to evaluate associations between physical activity and health parameters.

**Results:**

With ≤1 time per week as reference, exercising 2–3 times per week at baseline was associated with higher levels of VO_2max._ During follow-up, VO_2max_ increased with 2.8 mL ∙ kg^−1^∙ min^−1^ (95 % CI = 1.4, 4.3).

Women had more favorable body mass index (BMI), blood pressure, RHR and lipid profile than men. Total cholesterol, low density lipoprotein (LDL), RHR and diastolic blood pressure (dBP) were lower among participants who exercised 2–3 times per week or ≥4 times a week, compared with those with ≤1 time per week. Half of the participants reported increased daily PA during follow-up, with high intensity PA such as jogging by 8.6 min (SD 14.6) and 8.3 min (SD 18.2), among women and men, respectively. During follow-up dBP increased among men. Further, total cholesterol and LDL were reduced by 0.12 mmol/L and 0.13 mmol/L, respectively (95 % CI = −022, –0.01 and −0.22,–0.04).

**Conclusions:**

Exercise several times a week was associated with lower blood pressure and a favorable lipid status compared to lower weekly activity. During the 8-week follow-up of an employer initiated exercise program VO_2max_ increased, while total cholesterol and LDL were reduced.

**Trial registration:**

Current Controlled Trials ISRCTN13033050. Registered 21 August 2015.

## Background

“The London Transport Workers Study” from 1953 [1] was the first study that showed an association between lack of physical activity (PA) and increased risk of cardiovascular disease. From the 1980s studies reported that physical activity, partly based on self-reporting, prevented and improved lifestyle related disease [2, 3]. Later on, population studies have shown protective effects of PA on heart disease and cancer mortality [2, 4–7]. Activity of high intensity such as swimming, tennis and running had a better effect than low intensity activities [2, 3].

Recent prospective population studies with large number of participants have shown that low fitness is associated with increased all-cause mortality [8, 9]. A pooled data analysis of six prospective cohort studies, comprising in total more than 650 000 individuals [8], showed that 75 min of weekly brisk walking increased life expectancy after age 40 by almost two years, while one hour of daily similar PA increased life expectancy by 4.5 years [8]. These findings are in line with results from a subset of “the Oslo study” in which the men who were physically active almost every day had a 40 % lower all-cause mortality rate than those who were physically inactive [10].

There is a concern that sedentary work is detrimental to health and reducing sitting time at work is recommended [11]. Leisure time PA is known to have positive effects on fitness and cardiovascular function [12] and thereby should be a beneficial contributor to employees in various occupations. Increasing PA may affect blood pressure and blood lipids in a favorable way [13, 14]. Also, PA can induce enhanced glycemic control through lowering the HbA1c-levels in clinical settings [15]. Furthermore, PA has been shown to reduce markers of inflammation such as CRP [16, 17].

Both the World Health Organization and the European Union have launched large programs for workplace health promotion. These programs encourage employers to arrange for increased PA for their employees to improve health. Thus, lifestyle is no longer merely a private issue (http://www.who.int/occupational_health/topics/workplace/en/, http://www.enwhp.org/the-enwhp.html). Interventions to increase PA in workers have been carried out successfully, both by using structured workout sessions [18] and by internet based motivation programs [19]. However, there are few studies concerning the effect of motivation to physical activity initiated by employer at the workplace on physiological health parameters, like blood pressure and blood lipids [20]. The objective of this study was to prospectively investigate the physiological health effects of an 8-week activity program initiated by the employer, in a group of road- and office employees who are partly exposed to dust and/or have sedentary work.

## Methods

### Study design and population

**Fig. 1:**
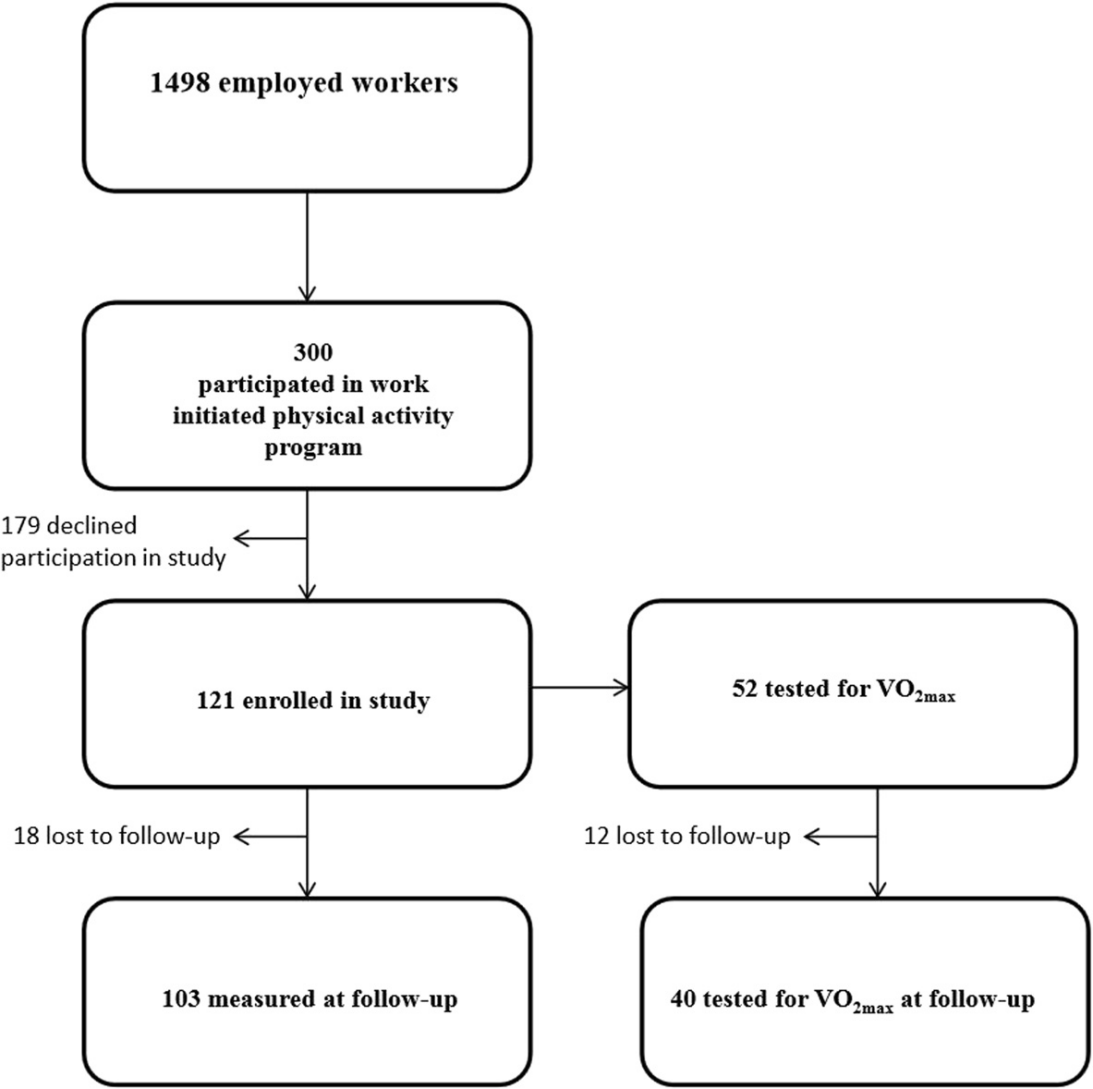
Flow diagram for the study

Out of 1498 employees in a Norwegian road maintenance enterprise, 89 % were men with a mean age of 45 years. Figure 1 most of the men were road workers (74 %). Information about the activity program was spread throughout the company via information meetings, the enterprise newspaper and the enterprise intranet for two months prior to its start up. All employees were invited to participate. Totally, 300 employees agreed to participate in the employer organized 8-week physical activity program (“Dytt®”), running from September 15^th^ to November 16^th^ 2014. The group of 300 included 73 % men of whom 38 % were road workers. The mean age of these men was 43 years.

Out of the 300, a subgroup of 121 individuals, 78 men (64 %) and 43 women with a mean age of 41.8 years (SD 12) and 42.6 years (SD 12.5), respectively, were enrolled in the present study on a voluntary basis. Among these, 86 % of the women and 53 % of the men reported college or university education (Table 1). All but two women were office workers, while 29 men (24 % of the total group) were road workers. The participants agreed to medical examinations including questionnaires, blood samples, blood pressure measurements and an aerobic fitness test before and after the program. Recruitment and baseline data collection was carried out two weeks prior to the start of the program, and follow-up data was collected within two weeks of ended program.

**Table 1:** Characteristics at baseline among females and males participating in the study

	Female (*N* = 43)^a^				Male (*N* = 78)^b^			
Outcome	Mean	SD	*N*	%	Mean	SD	*N*	%
Age (years)	42.6	12.5	.	.	41.8	12	.	.
Height (cm)	167.8	5.8	.	.	180.8	6.7	.	.
Weight (kg)	68.6	8.8	.	.	86	13.5	.	.
Body mass index (kg/m^2^)	24.4*	3.1	.	.	26.3	4	.	.
HbA1c (mmol/L)	5.2	0.3	.	.	5.3	0.4	.	.
Total cholesterol (mmol/L)	5.4	1.1	.	.	5.1	1.1	.	.
LDL (mmol/L)	3.2	0.9	.	.	3.2	0.9	.	.
HDL (mmol/L)	1.9*	0.4	.	.	1.4	0.3	.	.
CRP (mg/L)	1.9	1.8	.	.	1.9	2	.	.
Systolic blood pressure (mmHg)	115.5*	15.7	.	.	123.3	12.3	.	.
Diastolic blood pressure (mmHg)	74.2*	7.6	.	.	78.9	8.6	.	.
RHR (bpm)	67.4*	10.8	.	.	63.4	9.4	.	.
VO2max (mL ∙ kg^-1^∙ min^-1^)	33.5*	8.6	.	.	39.3	7	.	.
Leisure time physical activity (*n* ≤1 days/week)	.	.	16	37.2	.	.	29	37.2
Leisure time physical activity (*n* = 2–3 days/week)	.	.	21	48.8	.	.	36	46.2
Leisure time physical activity (*n* ≥ 4 days/week)	.	.	6	14	.	.	13	16.7
Smokers	.	.	0*	0	.	.	12	15.4
University/College	.	.	37*	86	.	.	41	52.6

*Significantly different between genders, *p* < 0.05. ^a^ *N* = 42 for weight, BMI, HbA1c and CRP; *N* = 41 for total cholesterol, LDL and HDL; *N* = 20 for VO_2max_. ^b^ *N* = 74 for HbA1c; *N* = 77 for total cholesterol, LDL, HDL and CRP; *N* = 32 for VO_2max_

Maximal oxygen uptake (VO_2max_) was only offered to those living in the eastern part of Norway, geographically close to Oslo. Thus, a subset of 52 among all participants (32 men and 20 women; 38 with college/university education) were included in this part of the study.

### Intervention

All subjects participating in the PA motivational program competed individually and in self-selected teams of 2–8 members in a virtual internet mountain track. Steps measured by a wrist-band accelerometer (Tappa®) were recorded on the personally designated profile on the competition website and both team and individual performances could be continuously monitored by participants throughout the program. Activities that were poorly measured by the wrist-band, such as swimming and spinning, could be converted into steps and entered manually. Weekly results were posted on the intranet pages of the enterprise. The best individual and team performance was rewarded at the end of the program.

### Physical activity and medical history

Questionnaires were completed at two occasions by the participants, prior to the medical examinations carried out at baseline and follow-up. All subjects’ anthropometrics such as height in cm (provided by the subjects themselves) and weight in kg was measured using a Sohenle® balance, medical history and number of days per week with PA were collected at both occasions. Self-reported PA was assessed by the question: *How often do you normally exercise*, with the response alternatives: *never*, *less than once per week*, *once per week*, *two to three times per week or almost every day* [21]. Answers were rearranged into: *once a week or less*, *2*–*3 times per week and 4 or more times per week*, *in order to obtain categories with a sufficient number of participants*. At follow-up, the number of steps were recorded from the online registration. Participants were asked if they had any change in low intensity- (e.g. walking) and high intensity activity (e.g. running) during the motivational program. Additionally, they were asked for, change in diet, alcohol consumption or smoking.

### Aerobic fitness

Aerobic fitness was determined by VO_2max_ and tested using a graded test on a cycle ergometer (Monark 874E, Monark Exercise AB, Vansbro, Sweden). The starting load was 70 W with a cadence of 70 revolutions per minute (RPM). Every minute the resistance was increased by 35 W until the subject was exhausted (cadence< 65RPM). Oxygen uptake was measured continuously with a Cosmed K4b^2^ breath by breath metabolismanalyser (CosmedSrl, Rome, Italy) and VO_2max_ was calculated from the highest 30 s averaging interval at the conclusion of the test.

### Blood pressure and resting heart rate

Blood pressure and resting heart rate (RHR) were measured on the left arm after five minutes of rest. The measurements were taken three times in intervals of one minute. The average of the last two measurements of both the systolic (sBP) and the diastolic pressure (dBP) was used in the statistical analysis. Blood pressure and RHR was measured with BpTRU® (Bp TRU medical devices, Coquitlam, Canada) on both occasions. All tests were performed using the same device and by the same researchers at the same time of the day both at baseline registration and at follow-up.

### Variables from blood analyses

Glycosylated hemoglobin (HbA1c) was collected in Ethylenediaminetetraacetic acid (EDTA) blood. Serum for investigation of lipids (cholesterol, low-density lipoprotein (LDL), high-density lipoprotein (HDL) and C-reactive protein (CRP)) was collected on gel tubes and then centrifuged 35 × 1000 rpm for 15 min within 60 min after the blood had been drawn from a vein. The tubes were sent by mail to the Department of Medical Biochemistry Oslo University Hospital and analyzed within 24 h.

HbA1c EDTA blood was analyzed with a Tosoh G7 HPLC analyser (Tosoh Bioscience, Inc. San Francisco, CA, USA) which use “high performance liquid chromatography” as the separation principle. The analytical variation is 1.7 %.

Cholesterol, LDL and HDL in serum were analyzed by enzymatic colorimetric method in the Cobas 8000. Analytical variation coefficients are respectively 3.0, 4.0 and 3.5 %.

CRP in serum was quantified by particle enhanced immunoturbidimetric method on Cobas 8000 (Cobas 8000 Modular Analyzer Roche Diagnostics, www.roche.com). Analytical variation is 8.0 %.

Blood was collected at the same time of the day on both occasions.

### Statistical analysis

Baseline gender differences were analyzed by linear regression adjusting for age and education. Mixed models were first applied to study the association between baseline PA and each of our outcome variables (HbA1c, total cholesterol, LDL, HDL, CRP, sBP, dBP, RHR and VO_2max_) collected at both baseline and follow-up. Second, mixed models were also applied to estimate the change in these health outcomes during follow-up. Third, we tested whether changes in exercise levels from baseline to follow-up had a significant effect on the changes in health outcomes. The mixed model analyses were adjusted for age, gender and education (completed college/university or not). The analysis of change in health outcomes were also adjusted for baseline PA. The mixed models were applied to the whole population and stratified according to gender. The effect of smoking was not significant (*p* >0.13 for all analyses), and was therefore not included in the analyses. A random intercept was added for subject. Further, we investigated whether the association between baseline PA and the health outcomes differed between genders, education groups or age levels. This was done by checking for significant interactions between baseline PA and each of these three covariates. Likewise, we examined interactions between time and each of these same covariates to determine if the adjusted change in outcome from baseline to follow-up differed between genders, education groups or age levels. All analyses were done in R (htttp://www.r-project.org).

## Results

### Loss to follow-up

Out of the 121 examined at baseline, 18 were not available at follow-up. Compared to the ones who remained in the study at follow-up, the 18 dropouts were younger (36.4 vs 43.1 years, *p* = 0.04) and had lower HDL (1.3 vs 1.6 mmol/L, *p* = 0.02) and higher CRP (3 vs 1.7 mg/L, *p* = 0.01). The dropouts tended to consist of more men and blue collar workers compared to those not lost to follow-up, but there were no differences of reported amount of exercise between dropouts and non-dropouts at baseline. In the subgroup of 52 who performed VO_2max_ at baseline, 13 did not perform this test at follow-up. These dropouts were younger than the ones who attended this examination at follow-up, 35.1 vs 44.8 years, *p* = 0.02.

### Baseline characteristics of the study population

Adjusted for age and education, females had a significantly, higher HDL (*p* <0.001), lower systolic blood pressure (*p* = 0.0026) and lower diastolic blood pressure (*p* = 0.0042) and higher RHR (*p*< 0.05) than males at baseline. Thirty seven (86 %) of the females and 41 (53 %) of the males had an education at college or university level. See Table 1.

Adjusted for age and gender, skilled male road workers (*N* = 29) had higher baseline values of RHR (4.4, *p* = 0.009) compared to office workers. Compared to those with college or university education, workers with lower education had higher BMI (2.3, *p*< 0.0001), RHR of 4.2 (*p* = 007) and HbA1c (0.16; *p* = 0.0001) when adjusted for age and gender.

### Physical activity

**Fig. 2:**
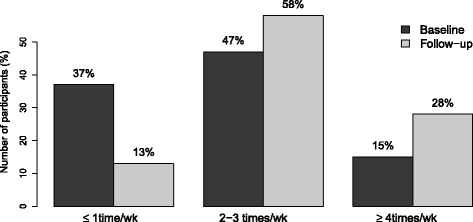
Change in weekly physical activity level between baseline and follow-up

According to the self-reported amount of exercise, 39 people exercised one day per week or less, 49 exercised 2–3 times per week and 15 at least 4 times a week at baseline. The corresponding figures at follow-up were 14, 60 and 29, respectively which was significantly changed compared to baseline (*p* = 0.001). See Fig. 2. There were no significant differences in baseline exercise level, nor in change in PA, between genders, educational levels or professional categories (office or road workers). Half of the participants reported to have increased PA frequency during follow-up, while only five persons reported a reduction in either low intensity or high intensity PA. According to what was self-reported in questionnaire, the mean increase in daily low intensity activity, such as walking, during follow-up was 13.7 min (SD 29.4) for men and 13.7 min (SD 17.2) for women. As for high intensity PA such as jogging, the corresponding figures were 8.3 min (SD 18.2) and 8.6 min (SD 14.6). Of the 103 participants observed at follow-up, 91 registered their steps at the Dytt® home page. The subjects had a median number of steps per day of 16 000 steps for the 8-week period but three subjects had registered less than 5000 steps.

### Association between activity levels and health outcomes

According to the mixed model we are able to use information both from baseline and follow-up. Thus baseline data of activity and outcomes at both measurements are used in the following presentation.

#### Maximal oxygen uptake

Compared to the reference category of exercise ≤ 1 day/week, reporting exercise frequency of 2–3 times per week at baseline was associated with higher levels of VO_2max_ for all subjects of 4.7 mL ∙ kg^−1^∙ min^−1^ (CI 0.5, 8.8; *p*= 0.029), and an exercise rate of 4 times or more per week showed similar results for men of 7.2 mL ∙ kg^−1^∙ min^−1^ (CI 0.6, 13.7; *p*= 0.033) taking values of both VO_2max_measurements into consideration. We found a relationship between reported amount of exercise and VO_2max_ (in relation to gender and age) for the 52 subjects with measured VO_2max_ at baseline. The association between high physical activity and improvement in VO_2max_ was most pronounced for young employees.

#### Diastolic blood pressure

Exercising 4 times a week or more at baseline had a favorable effect on reducing the dBP by 4.4 mm Hg (CI −8.1, −0.6; *p* = 0.024) when dBP was considered at both examinations. There was a significant interaction between baseline PA and gender, indicating that men seemingly responded better to high levels of physical activity. Compared to men exercising ≤ 1 time/week, men exercising more than 4 times a week had 6.2 mmHg (CI −10.9, −1.59; *p* = 0.0093) lower dBP. This difference in BP between low and high levels of physical activity was not shown in women.

#### Resting heart rate

Level of exercise equal to 2–3 times per week lead to a significant reduction in RHR of 4.3 bpm (CI −7.9, −0.5; *p* = 0.026) when RHR was considered at both occasions. Likewise, among those who exercised 4 or more times per week, RHR was reduced by 5.3 bpm (CI −10.4, −0.2; *p* = 0.042).

#### Total cholesterol

An exercise level of 2–3 times per week was associated with reduced cholesterol levels by 0.57 mmol/L (CI −0.97, −0.16; *p* = 0.0062) when all cholesterol values were considered. Furthermore, exercising 4 times per week or more also yielded cholesterol levels lower than the reference group by 0.63 mmol/L (CI −1.18, −0.08; *p* = 0.025).

#### Low density lipoprotein

Both exercise levels of 2–3 and 4 or more times per week revealed significant effects in reducing LDL levels of 0.47 mmol/L (CI −0.82, −0.11; *p* = 0.0096) and 0.52 mmol/L (CI −0.99, −0.04; *p* = 0.035), respectively. This was the case when LDL was considered at both baseline and follow-up.

Complete results for the association between baseline activity levels and health outcomes are shown in Tables 2, 3 and 4.

**Table 2:** Associations between blood parameters and exercise. Unstratified and stratified on gender

			Female (*n* = 43)				Male (*n* = 78)				All (*n* = 121)			
Outcome	Covariate	Category	β	95 % CI		*p*	β	95 % CI		*p*	β	95 % CI		*P*
HbA1c^c^	Exercise^a^	2–3 times/week	0.07	−0.07	0.22	0.32	0.12	−0.02	0.26	0.089	0.10	0.00	0.20	0.048
(mmol/L)		≥4 times/week	0.01	−0.20	0.22	0.94	0.06	−0.12	0.24	0.53	0.03	−0.10	0.17	0.62
	Age^b^		0.10	0.04	0.16	0.00095	0.16	0.10	0.21	*p* < 0.0001	0.14	0.10	0.18	*p* < 0.0001
	Gender	Female	.	.	.	.	.	.	.	.	−0.06	−0.16	0.04	0.25
	Education	College/University	−0.27	−0.48	−0.07	0.01	−0.15	−0.28	−0.03	0.017	−0.17	−0.28	−0.07	0.0012
Total	Exercise^a^	2–3 times/week	−0.48	−1.02	0.07	0.084	−0.59	−1.15	−0.03	0.038	−0.57	−0.97	−0.16	0.0062
cholesterol^d^		≥4 times/week	−0.61	−1.41	0.18	0.13	−0.64	−1.37	0.09	0.085	−0.63	−1.18	−0.08	0.025
(mmol/L)	Age		0.45	0.24	0.67	0.00014	0.12	−0.09	0.33	0.26	0.24	0.08	0.39	0.0028
	Gender	Female	.	.	.	.	.	.	.	.	0.15	−0.25	0.56	0.46
	Education	College/University	0.18	−0.59	0.95	0.64	0.15	−0.36	0.65	0.57	0.13	−0.29	0.54	0.54
LDL^e^	Exercise^a^	2–3 times/week	−0.38	−0.88	0.11	0.12	−0.49	−0.97	−0.01	0.044	−0.47	−0.82	−0.11	0.0099
(mmol/L)		≥4 times/week	−0.61	−1.33	0.12	0.097	−0.49	−1.12	0.14	0.13	−0.52	−0.99	−0.04	0.035
	Age		0.35	0.15	0.54	0.00095	0.07	−0.11	0.25	0.44	0.16	0.03	0.30	0.017
	Gender	Female	.	.	.	.	.	.	.	.	−0.12	−0.47	0.24	0.52
	Education	College/University	−0.02	−0.33	0.30	0.92	−0.05	−0.21	0.11	0.53	0.10	−0.26	0.46	0.59

^a^Reference value is exercise ≤1 times per week. ^b^age divided by 10. ^c^HbA1c had female *n* = 42, female obs = 76, male *n* = 74, male obs = 134, ^d^Total cholesterol had female *n* = 41, female obs = 77, male *n* = 77, male obs = 138. ^e^LDL had female *n* = 41, female obs = 76, male *n* = 77, male obs = 138

*HbA1c* = glycosylated haemoglobin level; *LDL* = low density lipoprotein

**Table 3:** Associations between blood parameters and exercise. Unstratified and stratified on gender

			Female (*n* = 43)				Male (*n* = 78)				All (*n* = 121)			
Outcome	Covariate	Category	β	95 % CI		*p*	β	95 % CI		*p*	β	95 % CI		*P*
HDL^c^	Exercise^a^	2–3 times/week	−0.02	−0.33	0.30	0.92	−0.05	−0.21	0.11	0.53	−0.05	−0.20	0.10	0.51
(mmol/L)		≥4 times/week	0.05	−0.41	0.51	0.82	0.01	−0.20	0.22	0.93	0.01	−0.19	0.21	0.94
	Age^b^		0.08	−0.05	0.20	0.22	0.04	−0.02	0.10	0.21	0.06	0.00	0.12	0.044
	Gender	Female	.	.	.	.	.	.	.	.	0.43	0.28	0.58	*p* < 0.0001
	Education	College/University	−0.11	−0.56	0.34	0.62	0.16	0.01	0.31	0.032	0.11	−0.05	0.26	0.17
logCRP^d^	Exercise^a^	2–3 times/week	−0.30	−0.81	0.20	0.23	0.00	−0.34	0.34	0.98	−0.10	−0.38	0.18	0.49
(mg/L)		> = 4 times/week	0.05	−0.69	0.78	0.9	−0.05	−0.50	0.41	0.84	0.00	−0.39	0.38	0.98
	Age^b^		−0.12	−0.32	0.08	0.25	0.10	−0.03	0.23	0.13	0.02	−0.09	0.13	0.68
	Gender	Female	.	.	.	.	.	.	.	.	0.07	−0.21	0.35	0.62
	Education	College/University	−0.16	−0.87	0.55	0.64	−0.27	−0.58	0.04	0.085	−0.23	−0.52	0.06	0.12
Systolic BP^e^	Exercise^a^	2–3 times/week	2.10	−6.54	10.75	0.63	1.08	−3.98	6.13	0.67	1.22	−3.18	5.62	0.58
(mmHg)		≥4 times/week	3.81	−8.77	16.39	0.54	−5.34	−12.06	1.37	0.12	−2.60	−8.60	3.40	0.39
	Age^b^		5.34	1.97	8.71	0.0027	5.38	3.46	7.31	*p* < 0.0001	5.58	3.91	7.24	*p* < 0.0001
	Gender	Female	.	.	.	.	.	.	.	.	−9.30	−13.67	−4.93	*p* < 0.0001
	Education	College/University	−6.49	−18.80	5.81	0.29	−0.85	−5.42	3.72	0.71	−1.77	−6.27	2.72	0.44

^a^Reference value is exercise ≤1 times per week, ^b^Age divided by 10, ^c^HDL had female *n* = 41, female obs = 77, male *n* = 77, male obs = 138, ^d^CRP had female *n* = 42, female obs = 77, male *n* = 77, male obs = 138. The CRP values was log transformed at analysis, ^e^Systolic BP had female *n* = 43, female obs = 78, male *n* = 78, male obs = 141

*HDL* = high density lipoprotein; *CRP* = *C*-reactive protein; *BP* = blood pressure

**Table 4:** Associations between blood parameters and exercise. Unstratified and stratified on gender

			Female (*n* = 43)				Male (*n* = 78)				All (*n* = 121)			
Outcome	Covariate	Category	β	95 % CI		*p*	β	95 % CI		*p*	β	95 % CI		*P*
Diastolic BP^c^	Exercise^a^	2–3 times/week	−1.20	−5.90	3.51	0.61	0.26	−3.24	3.76	0.88	−0.32	−3.09	2.45	0.82
(mmHg)		≥4 times/week	−0.64	−7.47	6.18	0.85	−6.24	−10.89	−1.59	0.0093	−4.35	−8.14	−0.57	0.024
	Age^b^		2.55	0.72	4.38	0.0077	2.45	1.12	3.79	0.00046	2.57	1.51	3.62	*p* < 0.0001
	Gender	Female	.	.	.	.	.	.	.	.	−5.83	−8.58	−3.08	*p* < 0.0001
	Education	College/University	−0.59	−7.26	6.09	0.86	−0.91	−4.08	2.26	0.57	−0.68	−3.51	2.15	0.63
RHR^d^	Exercise^a^	2–3 times/week	−5.50	−12.23	1.22	0.11	−3.72	−8.25	0.81	0.11	−4.25	−7.99	−0.51	0.026
(bpm)		≥4 times/week	−1.74	−11.51	8.02	0.72	−7.75	−13.76	−1.74	0.012	−5.31	−10.41	−0.20	0.042
	Age^b^		−1.05	−3.66	1.57	0.42	−1.94	−3.67	−0.22	0.028	−1.71	−3.13	−0.29	0.018
	Gender	Female	.	.	.	.	.	.	.	.	3.55	−0.1	7.27	0.061
	Education	College/University	2.95	−6.60	12.50	0.54	−4.62	−8.72	−0.52	0.028	−3.00	−6.82	0.82	0.12
VO_2max_^e^	Exercise^a^	2–3 times/week	4.68	−2.68	12.04	0.2	3.90	−1.27	9.07	0.13	4.67	0.50	8.83	0.029
(mL ∙ kg^−1^∙ min^−1^)		> = 4 times/week	−2.98	−13.79	7.84	0.57	7.19	0.64	13.74	0.033	3.77	−1.81	9.34	0.18
	Age^b^		−2.48	−5.54	0.57	0.1	−1.43	−3.38	0.52	0.14	−2.02	−3.64	−0.41	0.015
	Gender	Female	.	.	.	.	.	.	.	.	−6.49	−10.56	−2.42	0.0024
	Education	College/University	1.73	−9.62	13.08	0.75	4.29	−0.54	9.13	0.079	3.43	−1.07	7.93	0.13

^a^Reference value is exercise ≤1 times per week. ^b^Age divided by 10. ^c^Diastolic BP had female *n* = 43, female obs = 78, male *n* = 78, male obs = 141. ^d^RHR had female *n* = 43, female obs = 78, male *n* = 78, male obs = 141. ^e^VO_2max_ had female *n* = 20, female obs = 55, male *n* = 32, male obs = 55

*BP* = blood pressure; *RHR* = resting heart rate; *VO*_*2max*_ = maximal oxygen uptake

### Changes in health outcomes between baseline and follow-up

No changes in weight during follow-up were registered (results not shown). Four subjects had changed their tobacco consumption and two reduced their alcohol intake during follow-up.

#### Maximal oxygen uptake

**Fig. 3:**
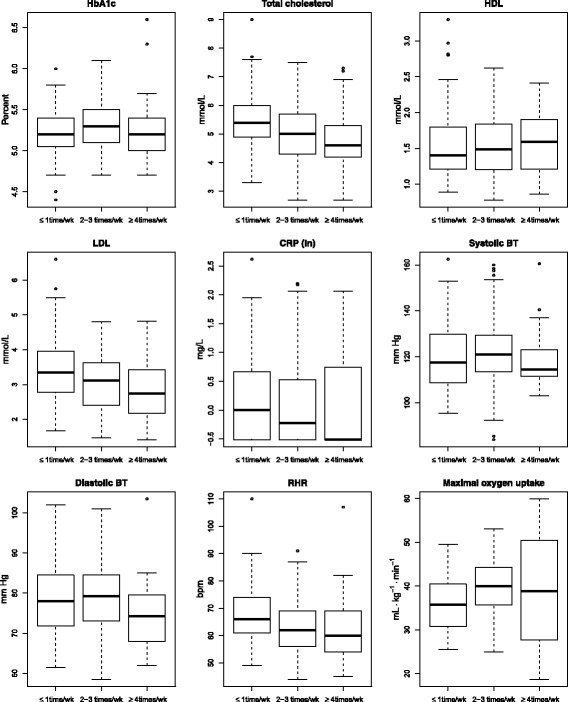
Differences in HbA1c, lipids, CRP, blood pressure, RHR and maximal oxygen uptake according to level of exercise, all measurements at both baseline and follow-up are included

VO_2max_ increased by 2.8 mL ∙ kg^−1^∙ min^−1^ (CI 1.4, 4.3; *p* = 0.00022) (Fig. 3).

#### Diastolic blood pressure

There was a significant increase of 1.67 mmHg (CI 0.23, 3.12; *p* = 0.024). The change in dBP differed significantly with education (*p* = 0.0068). Workers with low education showed an increase (4.4 mmHg; CI 2.03, 6.86; *p* = 0.0004) in blood pressure from baseline to follow-up. The highly educated workers did not have any significant change in blood pressure.

#### Total cholesterol

Cholesterol decreased from baseline to follow-up for all subjects of 0.12 mmol/L (CI −0.22, −0.01; *p* = 0.032). Changes in cholesterol differed significantly with educational level. While it increased (not significant) for workers with low education, it decreased significantly for those with high education by 0.21 (CI 0.08, 0.34; *p* = 0.0015).

#### Low density lipoprotein

LDL also showed a reduction between the two time points for females of 0.26 mmol/L (CI −0.38, −0.13; *p* = 0.00024) and for unstratified analyses 0.13 mmol/L (CI −0.22, −0.04; *p* = 0.0034). The change in LDL did also differ significantly with education. It increased (not significant) for the low education category, and decreased strongly for the high education category by 0.22 mmol/L (CI0.12, 0.32; *p* = 0.0001).

Table 5 shows the change in health outcomes between baseline and follow-up. Changes in PA from baseline to follow-up did not have any significant effect on changes in the different health outcomes.

**Table 5:** Change in parameters between baseline and follow-up. Total values and stratified on gender

	Female						Male						All					
Outcome	n	obs	β	95 % CI		*p*	*n*	obs	β	95 % CI		*p*	*n*	obs	β	95 % CI		*p*
HbA1c	42	76	−0.03	−0.08	0.02	0.19	74	134	−0.01	−0.05	0.03	0.53	116	210	−0.02	−0.05	0.01	0.16
Tot. chol.	41	77	−0.25	−0.42	−0.09	0.0043	77	138	−0.03	−0.17	0.11	0.68	118	215	−0.12	−0.22	−0.01	0.032
LDL	41	76	−0.26	−0.38	−0.13	0.00024	77	138	−0.05	−0.16	0.06	0.37	117	214	−0.13	−0.22	−0.04	0.0034
HDL	41	77	−0.03	−0.10	0.04	0.4	77	138	0.01	−0.03	0.05	0.62	117	215	0.00	−0.04	0.03	0.83
CRP^a^	42	77	−0.10	−0.35	0.15	0.4	77	138	−0.13	−0.35	0.09	0.24	118	215	−0.13	−0.29	0.03	0.11
Systolic BP	43	78	−1.63	−4.81	1.55	0.31	78	141	1.84	−0.80	4.47	0.17	120	219	0.53	−1.50	2.57	0.6
Diastolic BP	43	78	0.15	−2.14	2.44	0.89	78	141	2.58	0.72	4.44	0.0074	120	219	1.67	0.23	3.12	0.024
RHR	43	78	−1.56	−4.39	1.27	0.27	78	141	1.96	−0.11	4.02	0.062	120	219	0.68	−1.00	2.36	0.43
VO_2max_	20	55	2.08	0.23	3.94	0.03	32	55	3.47	1.38	5.55	0.0023	52	110	2.84	1.43	4.25	0.00022

^a^CRP values was log transformed at analysis

*HbA1c* = glycosylated haemoglobin level; *LDL* = low density lipoprotein; *HDL* = high density lipoprotein; *BP* = blood pressure; *RHR* = resting heart rate; *CRP* = *C*-reactive protein; *VO*_*2max*_ = maximal oxygen uptake]

## Discussion

The main results of this longitudinal study were twofold. First, we found that self-reported exercise two or more times per week at baseline was associated with more favourable blood pressure, RHR, total cholesterol and LDL levels. Second, we found an increase in VO_2max_ and decrease of total cholesterol and LDL during follow-up. Although half of the participants reported an increase in their activity level, we could not relate these results to changes in exercise levels.

### Physical activity and aerobic fitness

Among the 103 participants observed at follow-up, 91 registered their steps online. The median of registered steps was 16 000 steps per day, well above the recommended 10 000 steps [22]. This high level of PA during follow-up may partly be explained by a selection of fit individuals, but also an effect of the physical activity program since 50 % of the study participants reported an increase in their PA.

RHR was significantly lower among subjects who initially reported a high number of days of weekly exercise compared with sedentary ones. Individuals with RHR of more than 80 have an increased risk for cardiovascular disease and all-cause mortality compared to those with RHR lower than 60 [23]. Low RHR is favourable for myocard, and training with a high degree of intensity decrease the stiffness of the arteries and thus the strain on myocard [24]. RHR did not change during follow-up.

VO_2max_ had a mean increase of 2.8 mL ∙ kg^−1^∙ min^−1^ during follow-up. This is in line with a 10-week follow-up among sedentary office workers in Denmark where an increase in aerobic fitness of 1.45 mL ∙ kg^−1^∙ min^−1^was reported [19]. We could, however, not find a significant relationship between the increase in VO_2max_ and an increase in self-reported amount of activity. This relationship is found in studies with higher level of control over the PA carried out by participants and increased VO_2max_ values have been reported in these studies [18, 25]. Thereby, we have reasons to believe that our results reflect increased PA in the cohort.

### Physical activity and blood pressure

Our results showed that men seem to profit more strongly from a high weekly level of PA in reducing dBP as compared to women. For the total group dBP was lower among those with frequent PA compared with those who reported low activity at the first registration.

This is in line with a study of subjects with metabolic syndrome that demonstrated high intensity activity to be more favourable than low activity in terms of decreasing the blood pressure [25]. Additionally, studies among normotensives show that exercise has a beneficial effect on blood pressure [26]. Lowering blood pressure in the general population has an effect on the overall risk for cardiovascular diseases [27].

### Physical activity and blood analysis

We found lower cholesterol and LDL-levels among those who reported to exercise at least twice a week compared with those who reported low activity at baseline. Furthermore, during follow-up, LDL and cholesterol levels decreased for the study population, with a strong reduction for those with higher education. As half the study participants reported increased levels of PA, it is plausible that a decrease in lipid levels is due to increased PA, even though we were not able to establish a link between individual increases in PA and individual reductions in lipid levels.

Our findings are in line with what have been reported in clinical studies and a meta-analysis of studies on individuals with dyslipidemia has shown that exercise interventions over a few weeks may lead to increasing HDL and reduction of cholesterol/LDL levels [7].

During follow-up, a surprising increase in dBP was detected, still only for those with lower education. The same phenomenon was revealed in another follow-up of four months among cleaners, according to the authors possibly due to high intensity physical exercise causing an overload of the cardiovascular system of workers with low cardiorespiratory fitness [18].

### Strengths and limitations of the study

A strength of the present study is its prospective design with a high participation frequency of 85 % at follow-up. As in a cross-sectional study, it provides an opportunity to study associations between baseline PA and important health parameters. More importantly, it assesses individual changes between baseline and follow-up, where the individuals serve as their own control so between-subject variation is removed. In particular, it gives the possibility to see if individual changes in health are linked to changes in PA, and thus could establish a more direct causal relationship between PA and health outcome.

Mixed models provide an adequate method for analysing longitudinal data. They are robust to dropout that is missing at random, flexible in the choice of variance structures, and utilise all available observations including those from participants with non-complete observations.

All tests were performed using the same devices and by the same researchers and the blood was collected at the same time of the day on both occasions. As for the VO_2max_, standard instructions were followed when conducting the maximal oxygen uptake procedure.

There was a clear self-selection of healthy, younger, highly educated and possibly highly motivated individuals into the study. Only 22 % of the cohort consists of road workers compared to 74 % in the company as such. At baseline, the male road workers tended to be less physically active and had more cardiovascular risk factors compared to office workers. Adjusted for age and gender, their RHR and CRP (log transformed) were significant higher. This group is exposed to harmful dust which can be detrimental to the cardiovascular system [28]. This is of concern since studies of manual workers show increasing mortality among those who are physically active at work, but not among those who exercise during leisure time [29, 30].

Furthermore, the participants lost to follow-up had higher CRP and lower HDL-levels, two possible risk factors for cardiovascular disease, compared to the ones who remained in the study. This could imply an even further selection bias towards a more healthy population being subjected to follow-up.

A bi-product of the activity program could be life-style changes. At follow-up 12 reported more beneficial nutritional habits, three increased quality of sleeping, four participants had either reduced or quit smoking/snuffing whereas two reported having reduced the intake of alcohol.

A possible misclassification of exercise level cannot be ruled out since exercise magnitude and intensity were based on self-reports. Even though self-reports are practical in determining dose-response relationships with study outcomes, some subjects may have problems recalling and averaging frequencies and durations for the previous week or month and so self-reports may lead to both under and overestimating PA [31]. Therefore, it is plausible that the lack of statistical associations between self-reported PA and health parameters at follow-up was a result of the lack of a more precise instrument to measure PA. Still, one should not underestimate the setting used in this study; a “real world” intervention carried out by the enterprise as such. This means that the set-up, recruitment of participants and results are more in line with what can be achieved by employers who want to increase their employees’ PA. Stronger associations between exercise and health outcome effects may be found in laboratory or by researcher initiated interventions, but is difficult to apply in an occupational setting. This study may therefore provide a more realistic picture of how an actual physical activity intervention can affect health outcomes.

## Conclusions

An 8-week follow-up study of an employer initiated PA program for employees in road maintenance showed that regular PA is associated with a favourable lipid profile and blood pressure level.

### Ethics approval and consent to participate

The study was approved by the Regional Ethics Committee in Oslo (2014/1521). All participants were informed about the study and gave their written consent to participate.

### Consent for publication

Not applicable.

### Availability of data and materials

Not applicable.

## Abbreviations

BMI: body mass index
CRP: C-reactive protein
dBP: diastolic blood pressure
HbA1c: glycosylated hemoglobin
HDL: high density lipoprotein
LDL: low density lipoprotein
PA: physical activity
RHR: resting heart rate
sBP: systolic blood pressure
VO_2max_: maximal oxygen uptake

## References

[1] Morris JN, Heady JA, Raffle PA, Roberts CG, Parks JW (1953). Coronary heart-disease and physical activity of work. Lancet.

[2] Paffenbarger RS, Hyde RT, Wing AL, Hsieh CC (1986). Physical activity, all-cause mortality, and longevity of college alumni. N Engl J Med.

[3] Manson JE, Nathan DM, Krolewski AS, Stampfer MJ, Willett WC, Hennekens CH (1992). A prospective study of exercise and incidence of diabetes among US male physicians. JAMA.

[4] Wei M, Kampert JB, Barlow CE, Nichaman MZ, Gibbons LW, Paffenbarger RS, Blair SN (1999). Relationship between low cardiorespiratory fitness and mortality in normal-weight, overweight, and obese men. JAMA.

[5] Sui X, LaMonte MJ, Laditka JN, Hardin JW, Chase N, Hooker SP, Blair SN (2007). Cardiorespiratory fitness and adiposity as mortality predictors in older adults. JAMA.

[6] Blair SN, Wei M, Lee CD (1998). Cardiorespiratory fitness determined by exercise heart rate as a predictor of mortality in the Aerobics Center Longitudinal Study. J Sport Sci.

[7] Leon AS, Sanchez OA (2001). Response of blood lipids to exercise training alone or combined with dietary intervention. Med Sci Sports Exerc.

[8] Moore SC, Patel AV, Matthews CE, de Berrington Gonzalez A, Park Y, Katki HA, Linet MS, Weiderpass E, Visvanathan K, Helzlsouer KJ (2012). Leisure time physical activity of moderate to vigorous intensity and mortality: a large pooled cohort analysis. PLoS Med.

[9] Vigen R, Ayers C, Willis B, DeFina L, Berry JD (2012). Association of cardiorespiratory fitness with total, cardiovascular, and noncardiovascular mortality across 3 decades of follow-up in men and women. Circ Cardiovasc Qual Outcomes.

[10] Holme I, Anderssen SA (2000). [Physical activity, smoking and mortality among men who participated in the Oslo studies of 1972 and. Tidsskr Nor Laegeforen.

[11] Owen N, Salmon J, Koohsari MJ, Turrell G, Giles-Corti B (2014). Sedentary behaviour and health: mapping environmental and social contexts to underpin chronic disease prevention. Br J Sports Med.

[12] Oja P, Titze S, Kokko S, Kujala UM, Heinonen A, Kelly P, Koski P, Foster C (2015). Health benefits of different sport disciplines for adults: systematic review of observational and intervention studies with meta-analysis. Br J Sports Med.

[13] Semlitsch T, Jeitler K, Hemkens LG, Horvath K, Nagele E, Schuermann C, Pignitter N, Herrmann KH, Waffenschmidt S, Siebenhofer A (2013). Increasing physical activity for the treatment of hypertension: a systematic review and meta-analysis. Sports Med.

[14] Hagner-Derengowska M, Kałużny K, Hagner W, Plaskiewicz A, Bronisz A, Borkowska A, Budzyński J. Effects of Nordic Walking and Pilates exercise programs on blood glucose and lipid profile in overweight and obese postmenopausal women in an experimental, nonrandomized, open-label, prospective controlled trial. Menopause (New York, NY) 2015. Climacteric. 2015;18(6):835-40. doi:10.3109/13697137.2015.1058354 10.1097/GME.0000000000000446 25803666

[15] Lieber BA, Taylor B, Appelboom G, Prasad K, Bruce S, Yang A, et al. Meta-analysis of telemonitoring to improve HbA1c levels: Promise for stroke survivors. J Clin Neurosci. 2015;22(5):807–11. 10.1016/j.jocn.2014.11.009 25791996

[16] Ridker PM (2003). Cardiology Patient Page. C-reactive protein: a simple test to help predict risk of heart attack and stroke. Circulation.

[17] Beavers KM, Brinkley TE, Nicklas BJ (2010). Effect of exercise training on chronic inflammation. Clin Chim Acta.

[18] Korshoj M, Lidegaard M, Skotte JH, Krustrup P, Krause N, Sogaard K, Holtermann A (2015). Does aerobic exercise improve or impair cardiorespiratory fitness and health among cleaners? A cluster randomized controlled trial. Scand J Work Environ Health.

[19] Andersen LL, Sundstrup E, Boysen M, Jakobsen MD, Mortensen OS, Persson R (2013). Cardiovascular health effects of internet-based encouragements to do daily workplace stair-walks: randomized controlled trial. J Med Internet Res.

[20] Groeneveld IF, Proper KI, van der Beek AJ, Hildebrandt VH, van Mechelen W (2010). Lifestyle-focused interventions at the workplace to reduce the risk of cardiovascular disease--a systematic review. Scand J Work Environ Health.

[21] Moholdt T, Wisloff U, Lydersen S, Nauman J (2014). Current physical activity guidelines for health are insufficient to mitigate long-term weight gain: more data in the fitness versus fatness debate (The HUNT study, Norway). Br J Sports Med.

[22] Tudor-Locke C, Bassett DR (2004). How many steps/day are enough? Preliminary pedometer indices for public health. Sports Med.

[23] Saxena A, Minton D, Lee DC, Sui X, Fayad R, Lavie CJ, Blair SN (2013). Protective role of resting heart rate on all-cause and cardiovascular disease mortality. Mayo Clin Proc.

[24] Hanssen H, Nussbaumer M, Moor C, Cordes M, Schindler C, Schmidt-Trucksass A (2015). Acute effects of interval versus continuous endurance training on pulse wave reflection in healthy young men. Atherosclerosis.

[25] Tjonna AE, Lee SJ, Rognmo O, Stolen TO, Bye A, Haram PM, Loennechen JP, Al-Share QY, Skogvoll E, Slordahl SA (2008). Aerobic interval training versus continuous moderate exercise as a treatment for the metabolic syndrome: a pilot study. Circulation.

[26] Whelton SP, Chin A, Xin X, He J (2002). Effect of aerobic exercise on blood pressure: a meta-analysis of randomized, controlled trials. Ann Intern Med.

[27] Lewington S, Clarke R, Qizilbash N, Peto R, Collins R (2002). Age-specific relevance of usual blood pressure to vascular mortality: a meta-analysis of individual data for one million adults in 61 prospective studies. Lancet.

[28] Miller MR, Shaw CA, Langrish JP (2012). From particles to patients: oxidative stress and the cardiovascular effects of air pollution. Future Cardiol.

[29] Clays E, Lidegaard M, De Bacquer D, Van Herck K, De Backer G, Kittel F, de Smet P, Holtermann A (2013). The Combined Relationship of Occupational and Leisure-Time Physical Activity With All-Cause Mortality Among Men, Accounting for Physical Fitness. Am J Epidemiol.

[30] Holtermann A, Marott JL, Gyntelberg F, Sogaard K, Suadicani P, Mortensen OS, Prescott E, Schnohr P (2012). Occupational and leisure time physical activity: risk of all-cause mortality and myocardial infarction in the Copenhagen City Heart Study. A prospective cohort study. BMJ Open.

[31] Ainsworth B, Cahalin L, Buman M, Ross R (2015). The current state of physical activity assessment tools. Prog Cardiovasc Dis.

